# Analysis of the relationship between periodontitis and osteoporosis/fractures: a cross-sectional study

**DOI:** 10.1186/s12903-021-01496-1

**Published:** 2021-03-17

**Authors:** Seok-Jin Hong, Byoung-Eun Yang, Dae-Myoung Yoo, Sung-Jae Kim, Hyo-Geun Choi, Soo-Hwan Byun

**Affiliations:** 1 Research Center of Clinical Dentistry, Hallym University Clinical Dentistry Graduate School, Chuncheon, 24252 Korea; 2 Department of Otorhinolaryngology-Head and Neck Surgery, Hallym University College of Medicine, Dongtan, 18450 Korea; 3 Department of Oral and Maxillofacial Surgery, Dentistry, Hallym University College of Medicine, Anyang, 14068 Korea; 4 Hallym Data Science Laboratory, Hallym University College of Medicine, Anyang, Korea; 5 Department of Orthopaedic Surgery, Hallym University College of Medicine, Dongtan, 18450 Korea; 6 Department of Otorhinolaryngology-Head and Neck Surgery, Hallym University College of Medicine, Anyang, 14068 Korea

**Keywords:** Periodontitis, Osteoporosis, Fracture, Systemic inflammation

## Abstract

**Background:**

Chronic periodontitis is a multifactorial inflammatory disease resulting in patients exhibiting high levels of inflammatory factors causing systemic inflammatory bone destruction that may lead to osteoporosis development. The association between periodontitis and osteoporosis has been documented; however, the findings remain unclear. This study aimed to identify the association between periodontitis and osteoporosis using a cross-sectional study design and Korean Genome and Epidemiology Study (KoGES) health examinee data.

**Methods:**

This cross-sectional study used epidemiological data from the KoGES during 2004–2016. Of 125,324 participants (age, 40–79 years), 9969 with periodontitis and 115,332 controls (without periodontitis) were selected. We analyzed the history of osteoporosis and fractures of all participants. All participants were examined according to age, sex, income group, obesity, smoking habits, alcohol consumption, and food intake. To analyze the odds ratio (OR) of periodontitis for those with osteoporosis and fractures, a logistic regression model was used.

**Results:**

The adjusted odds ratio (aOR) of periodontitis for osteoporosis was 2.16 (95% confidence interval [CI], 2.01–2.31; *P* < 0.001). The aOR of periodontitis for any fracture was 1.54 (95% CI 1.46–1.62; *P* < 0.001).

**Conclusion:**

Osteoporosis and fractures are associated with periodontitis. Performing regular oral hygiene and examinations of bone mineral density are recommended to prevent aggravation of osteoporosis and periodontitis.

## Background

Periodontitis is a multifactorial inflammatory disease [1], and is the one of the most prevalent diseases involving the loss of periodontal tissues [2]. Periodontal disease in the elderly has become an important public health issue and a burden on the public health system [3]. Periodontitis has been considered a worldwide pandemic that results in reduced quality of life and disability [3]. Early studies reported that *Tannerella forsythia*, *Actinobacillus actinomycetemcomitans*, and *Porphyromonas gingivalis* are the primary causative agents in periodontal disease [4]. The microbial community in the oral cavity is referred to as the oral microbiota, oral microflora, or oral microbiome [5, 6]. They are present in the oral cavity and are mainly found in dental plaque [7, 8]. Patients with chronic periodontitis have high levels of inflammatory factors, such as tumor necrosis factor-α, interleukin-1, and interleukin-6 [9]. These inflammatory cytokines activate bone destruction by up-regulating the receptor activator of nuclear factor-κB ligand (RANKL) [9]. Previous studies revealed that the inhibition of RANKL could increase bone volume and density [10]. Based on these reports, chronic periodontitis and systemic inflammatory bone destruction could be related to the progression of osteoporosis. Bone minerals have the ability to promote inflammation and affect periodontal disease, and are considered to be an initiating factor in subsequent alveolar bone loss and disease progression [11].

Osteoporosis is considered a metabolic disease with an approximately 30% prevalence in women and 12% prevalence in men [12]. Additionally, the Nutrition and Health Survey in Taiwan (2005–2008) reported that the rates of osteoporosis for men and women older than 50 years were 23.9% and 38.3%, respectively [13]. Osteoporosis is a systemic disease that decreases bone mineral density (BMD). Osteoporosis is defined as a physiological process in which patients show bone structure defects. This can cause a higher risk of bone fractures and increase bone fragility [14]. The probability of fractures for patients could increase up to approximately 40% with osteoporosis, and the loss of autonomy leads to decreased quality of life [10, 15]. Early detection of osteoporosis may prevent fractures in patients with osteoporosis.

The BMD T-score is considered the essential standard for evaluating whether patients have osteoporosis. Femoral neck BMD was the reference standard for osteoporosis diagnosis, as assessed by dual-energy X-ray absorptiometry, using the Third National Health and Nutrition Examination Survey reference database from women aged 20–29 years [16, 17]. BMD is also the best tool for the fracture risk assessment. A 1-standard deviation decrease in BMD increases the fracture risk by 1.5–2.0-fold [18]. BMD is measured at several skeletal sites, but the lumbar and femoral regions are the most widely used in clinical practice [19, 20]. Hormone changes, especially decreased estrogen levels, are related to menopause and oxidative stress and can lead to bone loss, which can cause the development of osteoporosis [21].

Although periodontal disease is believed to be a localized disease, osteoporosis is a systemic process. Nevertheless, bone loss is a common characteristic in both diseases and is affected by many factors [22]. Osteoporosis and periodontitis show cumulative rates of bone loss with aging and have some common risk factors, including smoking habits, alcohol consumption, diabetes, and socioeconomic status [23, 24]. Decreased BMD may be related to periodontal tissue destruction. Previous studies have revealed that postmenopausal women with osteoporosis are susceptible to dental plaque development. Further, a recent study reported that osteoporosis can be diagnosed with a panoramic radiograph of women with pathologic bone fractures using radiomorphometric measurements, such as the mandibular cortical index. The association between periodontitis and osteoporosis/fractures has been documented; however, the findings remain unclear and there is no study focused on Asian or Korean individuals concerning this topic. This lack of adequate information might be due to differences in sample sizes, types of groups, and methods [25]. The aim of this study was to identify the association between periodontitis and osteoporosis and fractures using a cross-sectional study design and considering the Korean Genome and Epidemiology Study (KoGES) health examinee (HEXA) data.

## Materials and methods

### Study population and data collection

The Ethics Committee of Hallym University (2019-02-020) approved this study and the use of the KoGES HEXA data. All methods were carried out in accordance with relevant guidelines and regulations of the Ethics Committee of Hallym University. The requirement for written informed consent was waived by the Institutional Review Board. This cross-sectional study relied on data from the KoGES from 2004–2016. A detailed description of these data was provided in a previous study [26]. Among the KoGES Consortium, we used the KoGES HEXA data, which include information collected from urban residence participants 40 years or older. The baseline information was obtained from the 2004–2013 data were used.

### Participant selection

**Fig. 1:**
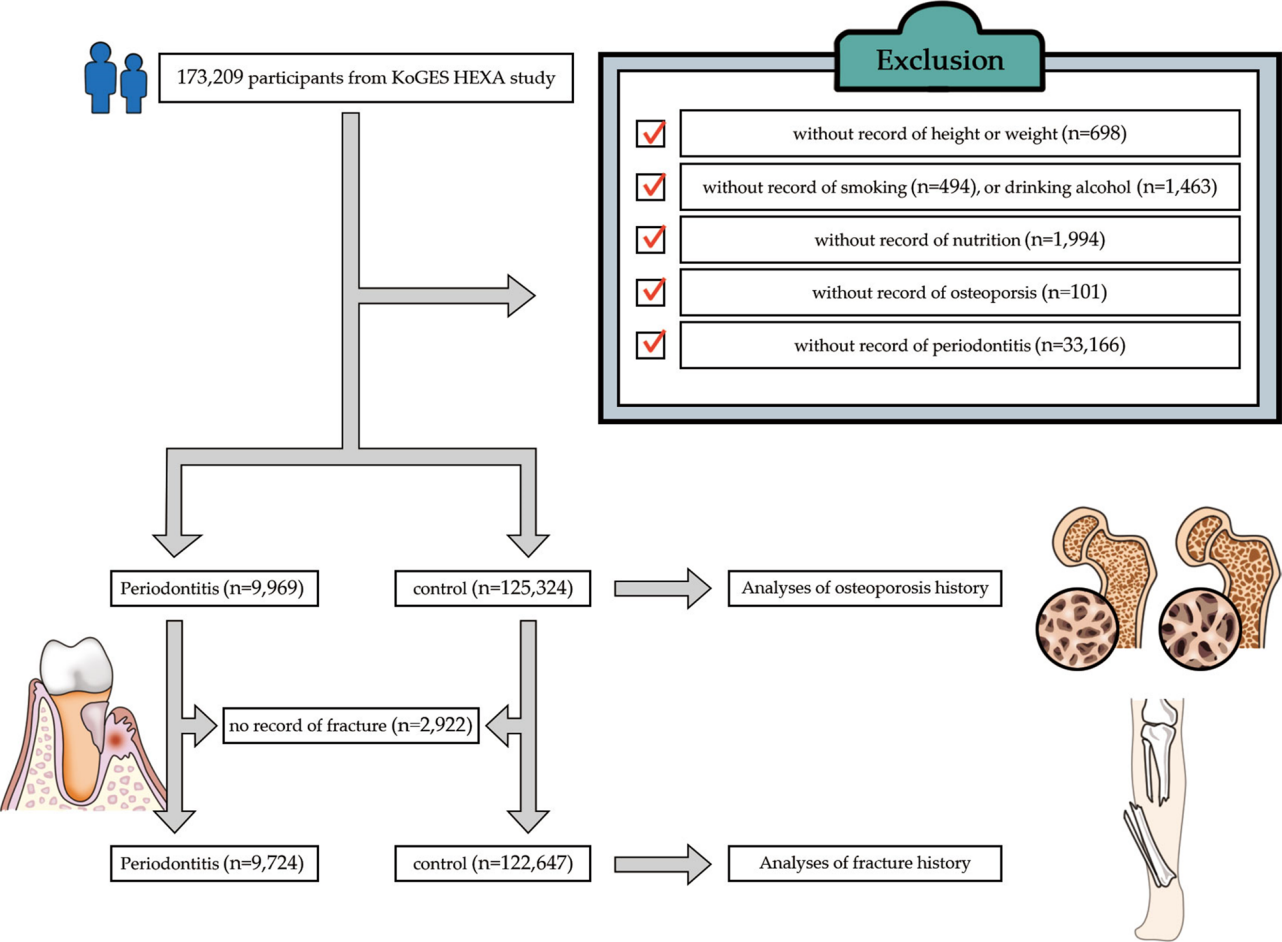
A schematic illustration of the participant selection process used for the present study. Of a total of 125,324 participants, 9969 with periodontitis and 115,332 controls (without periodontitis) were selected

Of 173,209 participants, we excluded those who did not have records pertaining to height or weight (n = 698), smoking history (n = 494), alcohol consumption (n = 1463), nutrition records (n = 1994), osteoporosis history (n = 101), and periodontitis (n = 33,166). Finally, 9969 participants with periodontitis and 125,324 control individuals (without periodontitis) were included (Fig. 1). Then, we analyzed the osteoporosis history of all participants in the periodontitis and control groups (primary objective). Thereafter, we analyzed the history of any fractures for those in the periodontitis and control groups (secondary objective). In total, 2922 participants were excluded from both groups because of a lack of records providing information on fractures.

### Survey

Trained interviewers interviewed the participants to obtain data regarding their history of periodontitis, osteoporosis, and fractures. Body mass index (BMI) was calculated as kg/m^2^ using the health check-up data. Smoking histories were used to create the following categories: non-smoker, < 100 cigarettes over the course of a lifetime; past smoker, quit more than 1 year ago; and current smoker [27, 28]. Alcohol consumption histories were used to create the non-drinker, past drinker, and current drinker categories. Nutritional intake (total calories [kcal/day], protein [g/day], fat [g/day], carbohydrate [g/day], calcium [mg/d], phosphorus [mg/d], and potassium [mg/d]) was surveyed using a food frequency questionnaire, as performed during a previous study [29, 30]. Income groups were categorized based on the household income as non-respondent, low income (approximately < $2000 per month), middle income (approximately $2000–$3999 per month), and high income (approximately ~ $4000 or more per month).

In this study, periodontitis was set as the independent variable, whereas osteoporosis and fracture were the dependent variables.

### Statistical analysis

Chi-squared tests were used to compare the roles of sex, income group, smoking, and drinking status. The independent t-test was used to compare age, BMI, and nutritional intake. Chi-squared and t-tests were used to evaluate distributional differences in categorical and continuous variables, respectively, between the participants with and without periodontitis.

To analyze the odds ratio (OR) of periodontitis for those with osteoporosis and fractures, multiple logistic regression models were used to evaluate the association between periodontitis (exposure) and osteoporosis or the presence of fractures (outcomes). The results are presented with the crude and adjusted models for age, sex, income group, BMI, smoking, alcohol consumption, total calories, protein, fat, carbohydrate intake, calcium, phosphorous, and potassium intake. During the subgroup analyses based on age and sex, the medians were fixed at 52 years or younger and 53 years or older.

Two-tailed analyses were conducted and *P* < 0.05 was considered statistically significant. The results were statistically analyzed using SPSS software (version 24.0; IBM, Armonk, NY, USA).

## Results

The general characteristics were different among participants in the periodontitis and control groups (Table 1). There were statistically significant differences in age, sex, BMI, income, smoking, alcohol consumption, total calories, fat levels, protein levels, and carbohydrate levels between the periodontitis and control groups (Table 1).

**Table 1:** General characteristics of participants

Characteristics	Total participants		*P* value
	Periodontitis	Control	
Age (mean ± SD)	54.8 ± 7.9	53.0 ± 8.3	< 0.001*
Sex (n, %)			< 0.001^†^
Men	3852 (38.6)	43,409 (34.6)	
Women	6117 (61.4)	81,915 (65.4)	
BMI (mean ± SD: kg/m^2^)	24.0 ± 2.9	23.9 ± 2.9	< 0.001*
Income (n, %)			< 0.001^†^
Missing, no response	766 (7.7)	10,854 (8.7)	
Lowest	3435 (34.5)	35,590 (28.4)	
Middle	3675 (36.9)	49,421 (39.4)	
Highest	2093 (21.0)	29,459 (23.5)	
Smoking status (n, %)			< 0.001^†^
Non-smoker	6685 (67.1)	91,121 (72.7)	
Past smoker	1796 (18.0)	18,593 (14.8)	
Current smoker	1488 (14.9)	15,610 (12.5)	
Alcohol consumption (n, %)			< 0.001^†^
Non-drinker	4784 (48.0)	64,037 (51.1)	
Past drinker	475 (4.8)	4537 (3.6)	
Current drinker	4710 (47.2)	56,750 (45.3)	
Nutritional intake (mean ± SD)			
Total calories (kcal/d)	1760.1 ± 580.5	1749.4 ± 569.4	0.073
Protein (g/d)	58.9 ± 26.6	59.8 ± 26.4	0.002*
Fat (g/d)	27.5 ± 18.5	28.3 ± 18.2	< 0.001*
Carbohydrate (g/d)	315.0 ± 95.2	310.0 ± 92.8	< 0.001*
Calcium (mg/d)	446.3 ± 264.2	447.3 ± 266.1	0.712*
Phosphorus (mg/d)	889.7 ± 369.5	896.4 ± 367.3	0.082*
Potassium (mg/d)	2243.9 ± 1095.1	2253.7 ± 1075.8	0.380*
Osteoporosis (n, %)	1255	7867	< 0.001^†^
Fracture (n, %)	1824	14,926	< 0.001^†^

SD, standard deviation; BMI, body mass index

^*^Independent *t* test

^†^Chi-squared test

The adjusted OR (aOR) of periodontitis for osteoporosis was 2.16 (95% confidence interval [CI], 2.01–2.31; *P* < 0.001) (Table 2). These results were consistent with the results of subgroup analyses, except for men 52 years or younger. The aORs were 1.98 (95% CI 1.69–2.33) for women 52 years or younger, 2.68 (95% CI 2.04–3.51) for men 53 years or older, and 2.12 (95% CI 1.96–2.30) for women 53 years or older (*P* < 0.001 for all).

**Table 2:** Crude and adjusted odds ratios (95% confidence interval) of periodontitis for osteoporosis

Characteristics	Osteoporosis	No osteoporosis	Odds ratios for osteoporosis			
	(n, %)	(n, %)	Crude	*P* value	Adjusted^†^	*P* value
Total participants (n = 135,293)						
Periodontitis	1255 (12.6)	7865 (6.3)	2.15 (2.02–2.29)	< 0.001*	2.16 (2.01–2.31)	< 0.001*
Control	8714 (87.4)	117,457 (93.7)	1.00		1.00	
Age ≤ 52 years, men (n = 21,513)						
Periodontitis	9 (0.6)	64 (0.3)	1.98 (0.98–3.98)	0.056	1.82 (0.90–3.68)	0.095
Control	1242 (99.4)	20,016 (99.4)	1.00		1.00	
Age ≤ 52 years, women (n = 45,451)						
Periodontitis	184 (7.2)	1457 (3.4)	2.21 (1.88–2.59)	< 0.001*	1.98 (1.69–2.33)	< 0.001*
Control	2370 (92.8)	41,440 (96.6)	1.00		1.00	
Age ≥ 53 years, men (n = 25,748)						
Periodontitis	69 (2.9)	260 (1.1)	2.61 (1.99–3.41)	< 0.001*	2.68 (2.04–3.51)	< 0.001*
Control	2350 (97.1)	23,069 (98.9)	1.00		1.00	
Age ≥ 53 years, women (n = 42,581)						
Periodontitis	994 (27.9)	6086 (15.6)	2.09 (1.93–2.26)	< 0.001*	2.12 (1.96–2.30)	< 0.001*
Control	2570 (72.1)	3232 (84.4)	1.00		1.00	

^†^Models adjusted for age, sex, income group, BMI, smoking, alcohol consumption, and nutritional intake (total calories, protein, fat, carbohydrate intake, calcium, phosphorous, and potassium intake)

Crude and adjusted odds ratios (95% confidence interval) were calculated by using multiple logistic regression analyses

The aOR of periodontitis for any fracture was 1.54 (95% CI 1.46–1.62; *P* < 0.001) (Table 3).

**Table 3:** Crude and adjusted odds ratios (95% confidence interval) of periodontitis for fracture

Characteristics	Fracture	No fracture	Odds ratios for fracture			
	(n, %)	(n, %)	Crude	*P* value	Adjusted^†^	*P* value
Total participants (n = 132,371)						
Periodontitis	1824 (18.8)	14,926 (12.2)	1.67 (1.58–1.76)	< 0.001*	1.54 (1.46–1.62)	< 0.001*
Control	7900 (81.2)	107,721 (87.8)	1.00		1.00	
Age ≤ 52 years old, men (n = 21,126)						
Periodontitis	295 (21.0)	2885 (14.6)	1.55 (1.35–1.77)	< 0.001*	1.50 (1.31–1.72)	< 0.001*
Control	1112 (79.0)	16,834 (85.4)	1.00		1.00	
Age ≤ 52 years old, women (n = 44,739)						
Periodontitis	312 (12.5)	3172 (7.5)	1.76 (1.55–1.99)	< 0.001*	1.65 (1.46–1.87)	< 0.001*
Control	2189 (87.5)	39,066 (92.5)	1.00		1.00	
Age ≥ 53 years old, men (n = 25,149)						
Periodontitis	475 (20.1)	3250 (14.3)	1.51 (1.36–1.69)	< 0.001*	1.47 (1.32–1.63)	< 0.001*
Control	187 (9.9)	19,537 (95.7)	1.00		1.00	
Age ≥ 53 years old, women (n = 41,357)						
Periodontitis	742 (21.5)	5619 (14.8)	1.57 (1.44–1.71)	< 0.001*	1.54 (1.41–1.68)	< 0.001*
Control	212 (78.5)	32,284 (85.2)	1.00		1.00	

^†^Models adjusted for age, sex, income group, BMI, smoking, alcohol consumption, and nutritional intake (total calories, protein, fat, carbohydrate intake, calcium, phosphorous, and potassium intake)

Crude and adjusted odds ratios (95% confidence interval) were calculated by using multiple logistic regression analyses

These results were consistent in all subgroups. The aORs were 1.50 (95% CI 1.31–1.72) for men 52 years or younger, 1.65 (95% CI 1.46–1.87) for women 52 years or younger, 1.47 (95% CI 1.32–1.63) for men 53 years or older, and 1.54 (95% CI 1.41–1.68) for women 53 years or older (*P* < 0.001 for all).

## Discussion

This study was based on the hypothesis that osteoporosis and fractures are likely associated with periodontitis. Our results revealed a statistically significant association between osteoporosis and periodontitis after adjusting for sex, age, income, obesity, smoking, alcohol consumption, and nutritional intake among the participants. Because this study was based on a questionnaire-based survey, it also analyzed fractures, and both osteoporosis and fractures showed similar results. The present study can be considered reliable because analyses of two different variables resulted in consistent outcomes. Only osteoporosis in younger men showed different results compared to the other subgroups. This could be explained by the lower cases of osteoporosis observed in younger men [31–33]. There were significant differences in the other subgroups of osteoporosis and periodontitis. These results would not confirm the interaction between age and osteoporosis in both sexes because of the lower number of younger men that participated in the study.

Some studies found that low BMD was related to the loss of alveolar bone and periodontal tissue [34–36]; however, others found no such association [37–39]. The influence of oral hygiene on the relationship between BMD and periodontal disease was investigated by previous studies with varying results [40–42]. Buffalo Women’s Health Initiative Observational Study demonstrated a statistically significant association between skeletal BMD and the clinical attachment level of postmenopausal women without subgingival calculus; however, no statistically significant association was found for postmenopausal women with subgingival calculus [41]. On the contrary, another study reported that participants with periodontitis who underwent regular dental care had a 1.3-fold risk of osteoporosis, but that the risk was sixfold higher for participants with periodontitis who did not undergo dental care. The study recommended regular check-ups for periodontitis patients to prevent aggravation related to osteoporosis [42]. Previous studies demonstrated that antiresorptive treatments, including hormone replacement therapy, antiresorptive agents, calcium, and vitamin D supplements, can have positive effects on the periodontal condition of postmenopausal women [43, 44].

The different conclusions of previous studies might have resulted from differences in the study designs, various sample sizes, and various influential factors. Most studies used a cross-sectional or retrospective design to demonstrate the association between osteoporosis and periodontitis [35–39, 44, 45]. A population-based cohort study revealed a statistically significant association between periodontitis and osteoporosis after adjusting for demographic characteristics [46]. However, the study did not include potential influential factors, such as systemic conditions or comorbidities. Additionally, there are very few studies on the association between osteoporosis and periodontitis in Asia, especially in Korea. Therefore, this study was performed with adjustments for many factors, analyzing both osteoporosis and fracture, with many Asian participants.

The pathophysiology between osteoporosis and periodontitis has also been explored by previous studies [47, 48], and various possible mechanisms might have been involved in the results. First, osteoporosis may induce alveolar bone loss in periodontitis [48]. Anbinder et al. suggested that periodontitis could be a risk factor for systemic bone loss, particularly for postmenopausal women [47]. Second, periodontitis is an inflammatory disease related to bacterial biofilm and immune response. Inflammatory cytokines and the subsequent immune responses are activated to protect periodontal tissue from bacterial invasion [9]. These cytokines could also contribute to the generation of osteoclasts from osteoclast progenitor cells and aggravate alveolar bone loss [9]. Monocytes induced by periodontal pathogens could induce systemic disease such as osteoporosis. Additionally, the interleukin-17/T-helper 17 pathway may be associated with RANKL-induced osteoclast formation in bone destruction. This mechanism may influence BMD [49–51].

This study used the data of a large population. Despite this advantage, there were a few limitations. First, it was impossible to include all influencing associative factors because the KoGES data did not include all confounding factors. For example, medical treatment and drug intake could be influential factors; however, they could not be included in this study. This study attempted to adjust for as many factors as possible to minimize surveillance bias. Second, the KoGES data were collected using a questionnaire survey; therefore, the accuracy of the survey used for this study could be questionable. Third, fractures may also occur because of trauma. However, the analysis of fractures did not have a great influence on this study because it was performed to complement the analysis of osteoporosis. Fourth, this was a comparative study with no evidence of a causal relationship. Fifth, it was based on a self-reported questionnaire. Self-reported questionnaires have the disadvantage of inaccuracy in diagnosis for periodontitis and osteoporosis/fracture. Therefore, this study could not present the severity and extent of periodontitis. Finally, the reliability of the questionnaires in terms of the frequency of smoking, frequency of alcohol consumption, and nutritional intake is unclear. To collect exact data, the reliability and validity of the questionnaire-based survey should be examined in future studies. However, this study provides a few meaningful results regarding periodontitis and osteoporosis and fractures. First, this study used a large sample population. Based on our investigation, this study is the largest population-based sample study of an Asian population. Second, the risk factors for osteoporosis and periodontitis increase with aging. Furthermore, women are at higher risk for osteoporosis than men, and women experiencing estrogen deficiency after menopause are at higher risk for osteoporosis [52, 53]. Additionally, obese individuals tend to have higher BMD than those with normal weight measurements [54]. This study considered nutritional intake, age, sex, obesity, income, smoking, and alcohol consumption as influential factors to evaluate the independent association between periodontitis and osteoporosis and fracture. Further studies should be conducted using a larger number of participants and with a clinician’s diagnosis, by using public health insurance data.

## Conclusions

This study demonstrated that osteoporosis and fractures are associated with periodontitis. Dentists and physicians should consider the possibility of interactions between osteoporosis and periodontitis. Furthermore, it is recommended that clinicians should perform examinations to determine whether the examined patients have osteoporosis and periodontitis. Additionally, regular oral hygiene and the BMD T-score examinations should be performed to prevent the aggravation of osteoporosis and periodontitis.

## Data Availability

The data that support the findings of this study are available from the database of Korean Genome and Epidemiology Study (KoGES) http://www.nih.go.kr/contents.es?mid=a40504010000 but restrictions apply to the availability of these data, which were used under license for the current study, and so are not publicly available. Data are however available from the authors upon reasonable request and with permission of Korean Genome and Epidemiology Study (KoGES).

## Abbreviations

aOR: Adjusted odds ratio
BMD: Bone mineral density
BMI: Body mass index
CI: Confidence interval
HEXA: Health examinee
KoGES: Korean Genome and Epidemiology Study
OR: Odds ratio
RANKL: Receptor activator of nuclear factor-κB ligand
SD: Standard deviation
